# Experience vs. Theory

**Published:** 1903-07

**Authors:** L. L. Loggins

**Affiliations:** Willis, Texas


For Texas Medical Journal.

### Experience vs. Theory.

BY L. L. LOGGINS, M. D., WILLIS, TEXAS.

"Commence with the experiment and then find out the reason" was the dogma of Da Vinci, and this is the rugged philosophy that has been rolling down the centuries since his day in the accomplishment of all that is good and practical. The men of history who have accomplished most in the various fields of thought, science and industry have been found foremost in the ranks of those who seek for results of a material character before looking to the glitter and hope of theory. After all, what is theory? We all know that it is but a conclusion that, in the end, may lead as well to disappointment as to success, to error as to truth. How often have we been carried away on the golden wings of a great theory, evolved by some greater medical light, and at last, at the bedside of a dying patient, been forced to acknowledge disappointment? How often has the medical world been startled by the announcement of a great discovery—a specific, a panacea—and as often seen it fade as noiselessly, but as certainly, as the disappearance of the rainbow? So often, in fact, that now we old fellows, at least, think but little of such announcements; nor do we take kindly to new things until statistics are piled up of a sufficient height to inspire confidence. We always stop to consider how very few remedies or drugs there are in existence that can claim the proud title of specific. Ah, how many are there of this class? Ever think of it?

It is common enough to call the old doctor a moss-back and a back number; but how often have we been called in consultation with the up-to-date young man and found him trembling like an aspen leaf over nothing very serious—a case of hysterics, perhaps?

Let us always pause to consider of this fact: That we are daily using remedies and methods that were in use hundreds of years ago. Let us remember that even in the field of surgery our progress is not so phenomenal as we sometimes think, if we eliminate the great auxiliary found in chloroform and ether. Ever think of this? Ever go back and ponder over the achievements of Ambrose Paré?

The truth is, we do not think enough; we do not look about us and read the lessons that Dame Nature is constantly laying before us. We are dazzled by the literature that commercialism is constantly holding up for our inspection, and forget that the world did exist before the unfolding of all that is so new—just out. We forget that we have among us still living and in active practice men who had healed the sick for years before the present day regimen burst upon the world. Why, now and then I read an article that causes me to wonder how it happened that I was born back in the forties and actually survived my birth along with my fortunate mother. But after sobering down long enough to consider that in the rural districts the old granny is still plying her vocation as midwife, surrounded by healthy mothers and ruddy-cheeked children, I cool down.

As a matter of course, medicine and surgery are advancing along with all things that are useful and beneficial to mankind; but I think it dangerous to be too eager to lay down old weapons and pick up new ones.

The other day I read an article on "Obstetrics," in which the writer (a man of national reputation) stated that it was criminal for a physician to fail to thoroughly wash out the uterus immediately after the birth of a child and for several days thereafter. This statement may be (doubtless will) be accepted as law and gospel by students and very young men; yet we older ones of the profession know that it is simply "bosh." We know that we should first be clean ourselves, be careful not to infect the body by carelessness in our manipulations; see that no clots are left; that the womb has not been left in a lax condition; and—well, this is about all. If we have done our duty in these respects nature will be obliging enough to do the rest. This same writer would not permit the parturient woman to sit up for two weeks. I believe that the bed is the place for such woman for two weeks—maybe longer; but after a few days I permit them to prop themselves against their pillows and rest themselves. This is pleasing to the ladies, and the position is beneficial. And about this time I am inclined to think an aseptic or antiseptic douche is not out of place. Of course the douche is called for at the very first appearance of anything like a septic condition. But how relatively seldom are such conditions seen? How many women have we assisted amid the most wretched and dirty surroundings and not the slightest symptom out of the normal noted?

Old women, and not a few doctors, constantly insist on giving the mother and child a dose of castor oil. What for? If we have had the mother under observation even for a few days, her bowels are all right; and, if not, we have probably moved by soapsuds. In any event, I never insist on the oil for the mother under twelve hours, and at the end of this time nature will generally have obviated its use. As for the baby, we all know—or ought to know—that nature has placed in the mother's breast a cathartic of potency—the colostrum. See that the nipple is clean and aseptic and put the baby to the breast at once. It will draw into its system the medicine nature placed there for it, and the reflex action arising from its nursing will cause the uterus to contract more firmly.

Many infants suffer for water, and I never forget to advise the giving of this essential to life at frequent intervals. But I warn against giving Castoria, soothing syrup, paregoric, etc., every time the baby cries. A few drops of whiskey in warm, sweetened water is more efficient than any of these in nine cases out of every ten, for the chances are it needs nothing.

I expect that, were he asked for a candid opinion, the editor of the "Red-Back" would say that he believed the majority of puerperal septicemia cases owe their origin to unclean and meddlesome midwives and physicians. I make this statement or suggestion because such is my own view of the matter, and because this same editor "Dan'els" never writes anything that does not convince the thoughtful reader that it is true; and, therefore, few, if any, of us hesitate in accepting as true (or worthy of trial) whatever he lays before us. That is why his journal is read with interest—because we wish to avail ourselves of the experience (not the untried theory) of competent and practical men. Only give us the experience and—if we are thoughtful workers—we shall be able to get at the theory. Tell us how you found a patient and how you treated him, and, taking up the result of your treatment, we shall most likely find out the why. Give us more such reports in the journals and we shall be better doctors, for we, being put upon our own resources as to the why, will think more of the matter; and whenever the human mind can be induced to think for itself much will be accomplished.

